# Radiation Induced Cavernomas in the Treatment of Pediatric Medulloblastoma: Comparative Study Between Proton and Photon Radiation Therapy

**DOI:** 10.3389/fonc.2021.760691

**Published:** 2021-10-11

**Authors:** S. Joy Trybula, Mark W. Youngblood, Hanna R. Kemeny, Jeffrey R. Clark, Constantine L. Karras, William F. Hartsell, Tadanori Tomita

**Affiliations:** ^1^ Division of Pediatric Neurosurgery, Department of Neurosurgery, Ann and Robert H. Lurie Children’s Hospital, Northwestern University Feinberg School of Medicine, Chicago, IL, United States; ^2^ Department of Radiation Oncology, Northwestern University Feinberg School of Medicine, Chicago, IL, United States

**Keywords:** medulloblastoma, cavernoma, external beam radiation, proton beam therapy, pediatric, radiotherapy

## Abstract

Radiation induced cavernomas among children with medulloblastoma are common following external beam radiation (XRT) treatment with either photon or proton beams. However, with the increased utilization of proton beam therapy over the last decade we sought to determine if there was any difference in the development or natural history of these cavernous malformations (CM) or CM-like lesions. We performed a retrospective analysis of 79 patients from 2003 to 2019 who had undergone resection of medulloblastoma and subsequent XRT (30 photon or 49 proton beam therapy). The average age of patients at radiation treatment was 8.7 years old. Average follow up for patients who received photon beam therapy was 105 months compared to 56.8 months for proton beam therapy. A total of 68 patients (86.1%) developed post-radiation CMs, including 26 photon and 42 proton patients (86.7% and 85.7% respectively). The time to cavernoma development was significantly different, with a mean of 40.2 months for photon patients and 18.2 months for proton patients (p = 1.98 x 10^-4^). Three patients, one who received photon and two who received proton beam radiation, required surgical resection of a cavernoma. Although CM or CM-like lesions are detected significantly earlier in patients after receiving proton beam therapy, there appears to be no significant difference between the two radiation therapy modalities in the development of significant CM requiring surgical resection or intervention other than continued follow up and surveillance.

## Introduction

Pediatric intracranial tumors are the most common solid malignancies in children, comprising nearly a quarter of all childhood cancers (1, 2). Medulloblastomas, a malignant embryonal tumor often affecting children aged 5-9 years old, constitute about 20% of pediatric CNS tumors (2, 3). In children greater than 3 years of age, standard of care consists of surgical resection, external beam radiation (XRT), and chemotherapy. Advancements in the characterization of the histologic subtypes of medulloblastoma have allowed for directed treatment by incorporating molecular pathogenesis in patient stratification for decision making (4). However, long term survival estimates range between 60-80%, and pediatric patients are often left with sequelae secondary to treatment (3, 5). Specifically, cranial radiation has been associated with a variety of neurocognitive, neuroendocrine, and neuro-vasculopathies in pediatric patients (6–9). Vascular complications, for example ischemic strokes, hemorrhage, or the development of vascular malformations such as telangiectasias or cavernous malformations have been described in the literature (10–15). As such, efforts to reduce radiation dosages or utilize alternative options, such as proton therapy, have been explored within the pediatric population (16–19). Proton beam therapy has emerged as a viable substitute given improved precision and reduction of toxicity to surrounding tissues (16, 20–22). Further, some studies have associated the use of proton therapy with neurocognitive preservation, reduced endocrinopathies, and even improved long-term intellectual outcomes compared to traditional photon treatment (16, 22–24).

The development of radiation induced cavernomas in the pediatric population following external beam radiation of medulloblastomas is well studied, however there remains a paucity of data surrounding the impact of proton therapy in the development of cavernous malformations (CM) or CM-like lesions. Given the promise of proton beam therapy as an alternative to traditional photon therapy, we sought to determine if there was any difference in the development or natural history of these CM or CM-like lesions between these treatment cohorts.

## Materials and Methods

We performed a retrospective review of 79 children with surgically resected medulloblastoma, who received postoperative XRT, and chemotherapy at our institution. All patients were diagnosed before 18 years of age and had over one year of MR imaging surveillance after RT. Data of 79 patients was collected on radiation treatment, clinical course, and the presence or absence of CM. Neuroimaging studies were reviewed for the presence, number, size, and anatomic location of dot-like cavernous malformation and CM. Gradient echo sequence (GRE) or susceptibility weighted imaging (SWI) were part of the established follow up imaging protocol intended to detect possible radiation induced late effect such as radiation-induced CM. The diagnosis of CM was done based on four-tier Zabramski classification; Type I-III are visible on T1-T2-weighted imaging, whereas Type IV, the dot-like cavernoma lesion is visualized only on GRE or SWI weighted images (25). Approval was obtained from the hospital’s institutional review board prior to the retrieval of clinical and radiographic data (IRB 2005-12692).

All patients were followed clinically and radiographically at different intervals beginning at 3 months postoperatively with follow up radiological examination repeated every 3 months for the first 2 years and then every 4-6 months until the 5^th^ year and then yearly depending on the status of the disease.

Statistical analysis and accompanying figures were generated using RStudio (Version 1.2.1280), with R version 3.5.3. Statistical significance was defined as p value < 0.05. For all independent variables (Sex, Diagnosis, Radiation Type) we assessed for associations with the potentially dependent variables (Formation of Cavernoma, Cavernoma Resection) *via* Chi-squared tests. Relationships were further assessed *via* Fisher’s exact tests for all possible values within the independent and dependent variables. The radiation type was also compared with time to cavernoma formation and patient age using the Student’s t-test. A Kaplan-Meier survival curve was generated to compare likelihood of remaining lesion free following each type of radiation treatment. Comparisons between the resulting curves were performed using the log-rank test.

## Results

**Figure 1:**
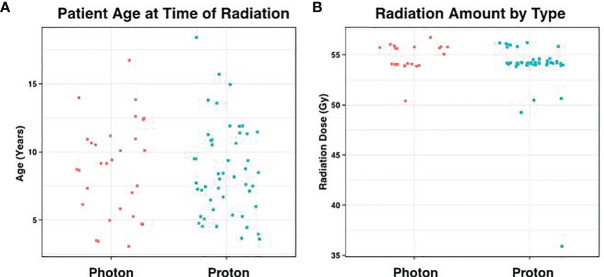
Distribution of Age and Radiation Dose. **(A)** Average patient age at the time of radiation was 8.7 years, which did not differ significantly according to radiation type (p = 0.79). **(B)** Similarly, the amount of posterior fossa radiation received between the two groups showed no statistical difference (p = 0.07).

Between 2003 and 2019, a total of 79 patients (51 male and 28 female) with medulloblastoma had surgical resection for their primary tumors at our institution. Median age was 8.7 years old (3.2 – 18.3years), which was similar for patients receiving photons and protons (mean = 8.9 vs 8.6 respectively; p = 0.79, **Figure 1A**). All cases received chemotherapy in addition to radiation. There were 30 patients that underwent traditional photon therapy, while 49 received proton beam therapy.

**Figure 2:**
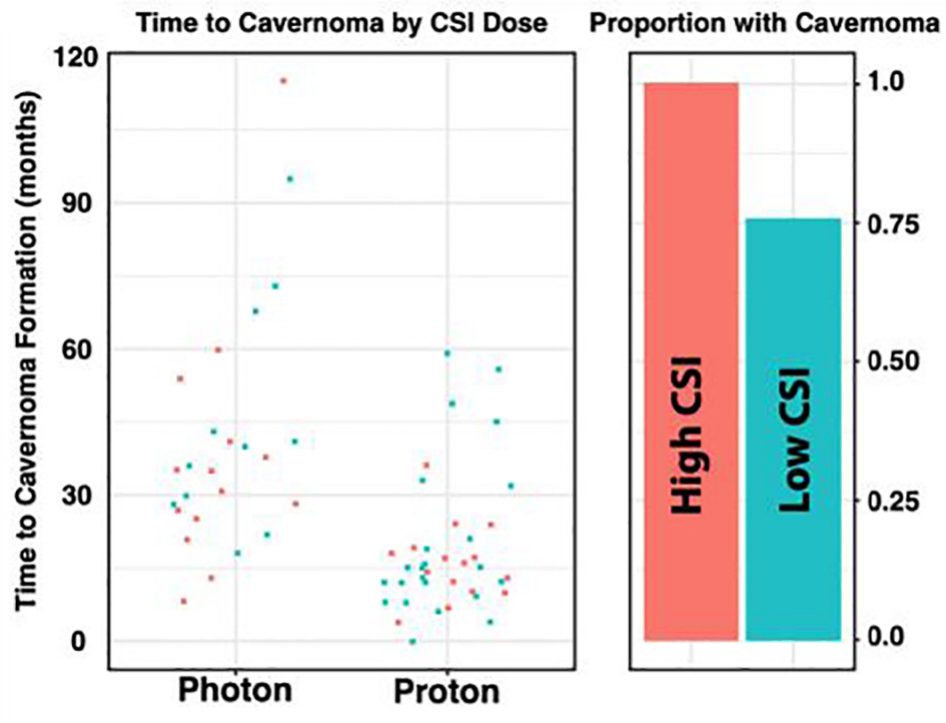
Distribution of Radiation Dose by Radiation Type. The photon and proton groups are divided into STANDARD (n=33) and HIGH (n = 28) dose CSI radiation, based on a threshold of 33. Among both the Photon and Proton groups, it does not appear that CSI dosage has an effect on the Time to Cavernoma formation (p = 0.49 and 0.29 respectively). Similarly, we do not observe a significant relationship between CSI dosage and formation of a cavernoma in the Photon or Proton groups (p = 0.99 and 0.77 respectively). However, when the groups of photon and proton were combined, a significant relationship between the CSI dosage and formation of CMs was noted (p =0.005).

In the photon group, the average total absolute posterior fossa radiation dose was 54.8 Gy, compared to an average dose of 54.2 Gy among the proton patients (p = 0.08; **Figure 1B**). We subsequently divided the photon and proton groups into standard (n=34) and high (n=29) dose CSI radiation (**Figure 2**); standard being 23.4 – 24 Gy and high being 36 Gy. These CSI doses were decided according to the patients’ risk factors per national medulloblastoma protocols (*i.e* COG A9961, ACNS0331, ACNS 0332, POG 9031). We had one patient in the photon group who received 30.6Gy and was included in the standard group for analysis. Another patient who had 41.1Gy photon radiation who was included in the high dose group. We assessed differences in formation of CMs and time to formation. Among both the photon and proton groups, CSI dosage did not effect on the Time to CM formation (p = 0.49 and 0.29, respectively). There was no significant relationship when all samples are considered (i.e. without regards to radiation type) (p = 0.75**)** **Figure 2**. There was also no significant relationship between CSI dosage and formation of CMs in the photon or proton groups (p = 0.99 and 0.77 respectively). When proton and photon groups were combined, a significant relation between the CSI dosage and formation of CMs is noted (p = 0.005); the higher CSI dosage was associated with increased risk of CM formation.

At the initial tumor presentation, preoperative MR of the brain did not reveal CM in any patients. From the time of radiation completion, regular surveillance imaging was performed *via* 1.5T or 3T MRI, and follow-up ranged from 9 to 185 months (mean 75 months). Cavernomas or CM-like lesions were noted on either gradient echo sequence (GRE) or susceptibility weighted imaging (SWI) based on our institutional MR protocol. We found no significant difference between proton or photon therapy and the MRI sequence used to detect CM or CM-like lesions. For patients who had their lesions detected by GRE sequence, we found on average 29.8 months from radiation to development of cavernoma compared to 23.7 months for patients who had their first lesion detected by SWI sequence.

**Figure 3:**
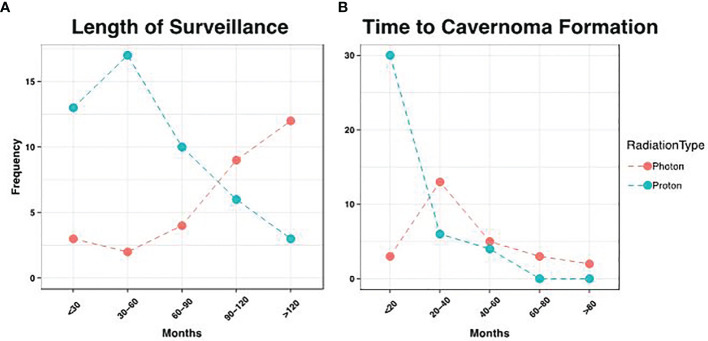
Distribution of Surveillance and Cavemoma Formation. **(A)** Patients that received photon therapy had longer length of surveillance relative to proton patients, consistent with more recent use of the latter modality (p= 7.28 x 10^-6^). **(B)** However, proton patients had earlier development of cavemomas, with an average time to formation of 18.1 months, vs. 40.2 months in photon patients (p = 2.01 x 10^-4^).

One patient died soon after radiation therapy who had less than 12 months follow-up. Nine patients had follow-up less than 2 years but were included in the analysis. Of these patients, 5 had no CMs detected on MRI. The average follow-up was 105 months for photon patients, but 56.8 months for proton patients (p = 7.65 x 10^-6^), consistent with more recent use of proton beam therapy (**Figure 3A**). We did not detect a statistical difference in the patient age at time of radiation between these groups (p = 0.79). There were three cavernomas that ultimately required surgical resection, though this was not statistically associated with sex (p = 1.0), diagnosis (p = 1.0), or XRT type (p = 1.0).

**Figure 4:**
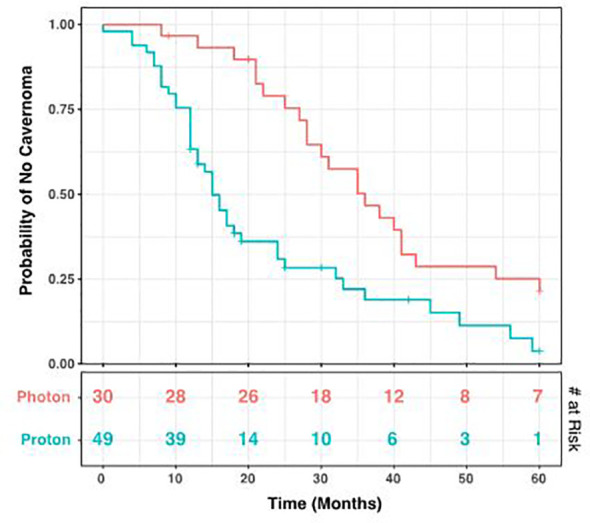
Kaplan-Meier Curve for Development of Cavernomas. Over a 60-month period after radiation, cavernoma development occurred earlier in patients receiving proton radiation compared to those receiving photons (log rank p= 5.95 x 10^-4^). The number at risk at each time point is shown in the bottom table. Tick marks indicate patients lost to follow-up.

**Figure 5:**
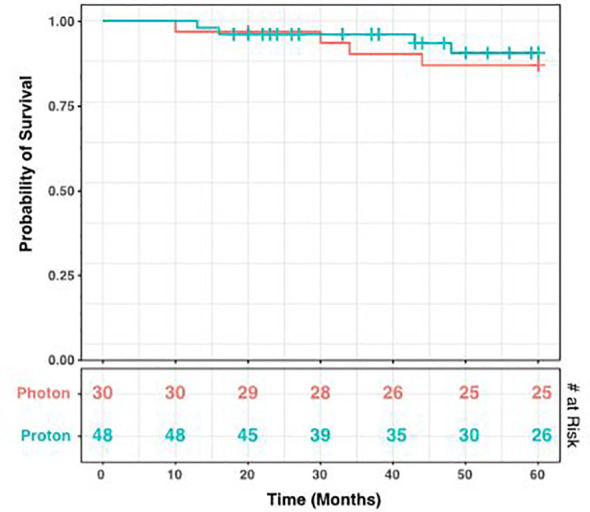
Kaplan-Meier Curve for Survival Stratified by Radiation Type. Over a 60-month period after radiation, there was no significant relationship detected between radiation type and mortality. The log rank test gives a p-value of 0.61. The number at risk at each time point is shown in the bottom table.

During the period of collected data, a total of 68 patients (86.1%) developed post-radiation CMs, including 26 photon and 42 proton patients (86.1% and 85.7% respectively). There was no statistical difference between these groups (p = 1.00), nor between CM formation and gender (p = 0.34). However, the time to CM development was significantly different, with a mean of 40.2 months for photon patients and 18.2 months for proton patients (p = 1.98 x 10^-4^, **Figure 3B**). Indeed, we observed significant differences in the Kaplan-Meier curves for photon and proton patients, such that proton patients tended to develop cavernomas earlier than photons (log rank p = 2.23 x 10^-4^). After 5 years of monitoring, 21.5% of photon patients remained free cavernomas, while only 3.3% of proton patients did (**Figure 4**). Additionally, when we evaluated overall survival stratified by radiation type, we found no significant relationship between photon or proton radiation and mortality (p=0.61) (**Figure 5**).

**Figure 6:**
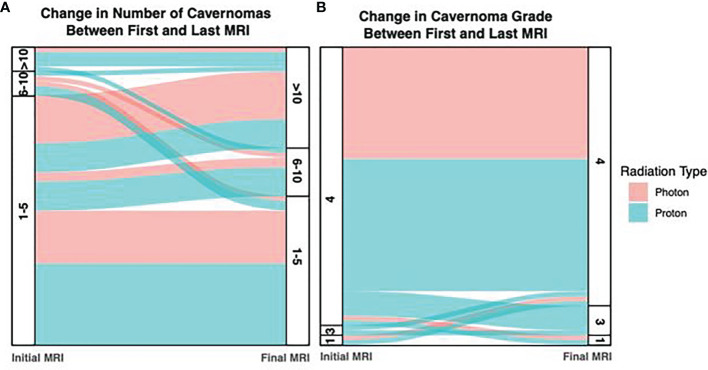
Alluvium Plot of Number of Cavernomas and Cavernoma Grade on Initial and Final Imaging. **(A)** The number of cavernomas detected increased between the initial and final surveillance imaging, however differences between the photon and proton groups were mild. **(B)** In photon patients, the number of grade 4 lesions (dot-like) were consistent over both imaging timepoints, however the number of grade 4 lesions decreased in proton patients, suggesting progression of some lesions.

For comparison between radiation types, the number of CMs or CM-like lesions identified on post-radiation MRI was bucketed into 1-5, 6-10, and >10 bins for each sample. On initial imaging, the distribution of CMs or CM-like lesions between these bins was similar for photon and proton patients, with 88.5% and 80.6% of patients respectively falling into the lowest bin (1-5 lesions). Notably however, four proton patients exhibited an elevated number of lesions (>10; 11.1%), while this occurred in only a single photon patient (3.4%). However, this trend was lost on the final follow-up imaging obtained for each patient, with 42.3% and 27.8% of photon and proton patients respectively falling into the largest bucket (**Figure 6A**).

CM or CM-like lesions were classified as Type IV lesions in 59 of 63 patients (93.6%), and this proportion did not significantly change at the time of last follow-up (55 of 63; 87.3%; p = 0.36; **Figure 6B**). Notably, patients that underwent proton radiation drove the small decrease in Type IV lesions at final follow-up, as these patients exhibited progression in some cases (92.1% Type IV initially, vs. 81.6% at time of last follow-up). By contrast, there was no change in the amount of Type IV lesions during the study period in patients who received photon radiation.

**Figure 7:**
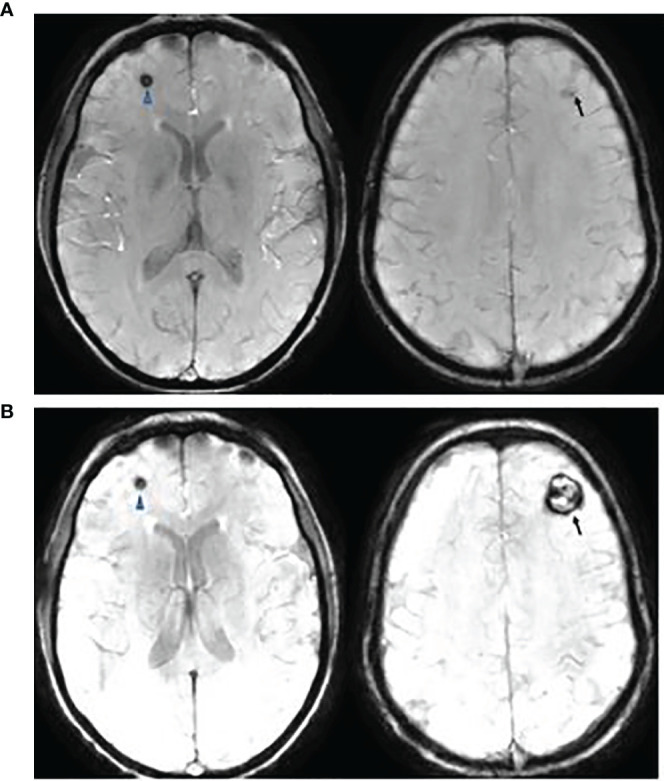
This is a 20-year-old female with medulloblastoma. She had a resection of anaplastic medulloblastoma followed by proton RT (36 Gy to CSI and 55.9 Gy to posterior fossa) and chemotherapy at the age of 10 years. Follow up axial Susceptibility-weighted Imaging, 8 years **(A)** and 10 years **(B)** after the therapy, showing interval increase of cavernyus angioma of the left frontal lobe (arrow) but a stable lesion in the right frontal lobe (arrowhead). She presented with increasing headaches which were resolved following a resection of left frontal cavernous angioma.

All CM or CM-like lesions were asymptomatic and detected on surveillance MRI, except for 3 patients: Two patients developed seizures (frontal and parietal lobe, respectively) and one presented with increasing headaches (frontal lobe, **Figure 7**). All were Type I lesions, confirmed to be cavernous malformation by surgical resection.

## Discussion

Traditional XRT following surgery has dramatically increased survival for medulloblastoma patients, however, it is associated with a variety of complications including endocrine dysfunction, stunted bone growth and development, neurocognitive deficits, secondary tumors and CM development (26–31). Arteries and capillaries are prone to radiation injury which directly disrupts the blood brain barrier causing edema and tissue hypoxia. Compared with photon therapy, proton beam therapy may enable similarly effective radiation regimens with less toxicity to nearby structures and may reduce the rate of secondary neoplasm development (32–36). It is understood that multiple *de novo* CMs may occur following conventional XRT (30). These *de novo* CMs are typically clinically silent and rarely result in symptoms requiring surgical intervention. Whether proton therapy differs from photon therapy in the risk for CM development is addressed by very few publications, however, given the potential for CMs to result in hemorrhage, seizure, and other neurologic deficits, elucidating the risk of cavernoma-genesis as it relates to radiotherapy modality has implications for clinical management and post-radiation surveillance protocols.

Lew et al. followed 59 pediatric patients for a mean of 7.2 years after medulloblastoma resection and subsequent XRT including CSI and chemotherapy (13). Patients received standard or hyperfractionated photon therapy or proton therapy and the study found no significant associations between CM development and radiation modality or dose. However, only four patients received proton therapy in their series and none of them developed CM. Eighteen (30.5%) patients in the study developed CMs with a median time of 6.1 years to lesion development following the conclusion of radiation therapy, and only one patient was symptomatic and required CM resection.

Grahovac et al. analyzed 37 pediatric patients for a mean of 5.2 years following medulloblastoma resection, XRT and chemotherapy treated at our institution earlier (12). While only 5 patients received proton therapy, 29 (78.3%) patients developed CM, 27 of whom developed only Type IV dot-like CMs in their series. The other two patients developed Type I CMs, one of whom was symptomatic with seizures and underwent CM resection. The latency interval between completion of RT and development of CM or CM-like lesions was 2.7 years.

These prior studies included both photon and proton beam radiation therapy, however lacked a sizable proton beam radiation cohort. In our study, we found that there was a statistically significant decrease in latency to develop CMs on imaging after proton therapy compared to after photon with a mean of 40.2 months for photon beam therapy and 18.2 months for proton beam therapy (p = 1.98 x 10^-4^). We also observed significant differences in the Kaplan-Meier curves for XRT and proton patients, such that patients receiving proton beam therapy tended to develop cavernomas earlier than those receiving photon therapy (log rank p = 2.23 x 10^-4^). This difference in proton and traditional photon therapy has not been previously documented in the literature. We had 3 patients who subsequently required surgical resection of their cavernoma. Two patients received proton therapy and one had received XRT. After 5 years of monitoring, 21.5% of photon patients remained free cavernomas, while only 3.3% of proton beam therapy patients did (**Figure 3**). Due to institutional experience and traditional use of photon therapy prior to proton beam therapy, we had much longer follow-up times for patients receiving photon therapy 105 months and 56.8 months, respectively.

In our cohort, we found no significant difference between standard and high dose CSI for time to CM formation in either proton or photon beam radiation group. However, we may have been underpowered to detect this difference. On the other hand, when combining proton and photon beam therapy together, there appears to be a statistically significant correlation between CM formation and increased radiation dose. Additionally, it appears that higher radiation dosage (> 36Gy) regardless of radiation type was correlated with increased development of CMs.

Proton therapy has been associated with a potentially higher risk of treatment-related morbidities in the pediatric population, namely the end-of-beam-path toxicity, which can cause radiation necrosis in structures treated near the tumor bed in the posterior fossa such as the brainstem and pons (37–39). In our cohort, we observed very few infratentorial CM compared with supratentorial CM and all of our symptomatic CM requiring surgery were located supratentorially. We found no pattern in the location of CM or CM-like lesions, however we may have been underpowered to detect this relationship. MR sequence and slice thickness can also affect the sensitivity to detect CM or CM-like lesions. In particular, SWI sequence has been shown to be more sensitive than GRE (12). In our study, we noted the first MRI sequence where a CM or CM-like lesion was detected and there was no significant difference between average time to formation of CM when evaluated by GRE or SWI sequence.

Zabramski’s classification has been used to describe cavernous malformations or CM-like lesions (25). Type 1 -III lesions reflect the time and degree of hemorrhage, but Type IV lesions are considered to be telangiectasia (12, 25).We found that the photon group had few changes in the four-tier Zabramski classification of CM or CM-like lesions between the first and last MRI compared to the proton beam radiation group. In the proton beam therapy group, fewer patients had Zabramski Type IV lesions on last MRI as noted in **Figure 4** suggesting progression of some of the CM or CM-like lesions. There was no significant difference between the two groups regarding symptomatic CMs or CMs requiring surgical intervention. Moreover, we found no significant difference for survival when stratified by radiation type This data is reassuring that proton beam radiation is safe, effective, and does not appear to increase the risk of developing symptomatic CMs. Earlier onset of CM or CM-like lesions may cause some alarm to clinicians, however surgical intervention can be avoided in most cases and these lesions should be observed along with primary disease surveillance. The underlying pathophysiology of these lesions is still unclear and further prospective studies should be performed to elucidate the inflammatory, vascular, and molecular drivers of these cavernous malformations and CM-like lesions.

## Data Availability Statement

The raw data supporting the conclusions of this article will be made available by the authors, without undue reservation.

## Author Contributions

ST, HK, and TT contributed to conception and design of the study. JC, ST, and HK organized the database. MY performed the statistical analysis. ST and HK wrote the first draft of the manuscript. CK, JC, TT, and WH wrote sections of the manuscript. All authors contributed to manuscript revision, read, and approved the submitted version.

## Conflict of Interest

The authors declare that the research was conducted in the absence of any commercial or financial relationships that could be construed as a potential conflict of interest.

## Publisher’s Note

All claims expressed in this article are solely those of the authors and do not necessarily represent those of their affiliated organizations, or those of the publisher, the editors and the reviewers. Any product that may be evaluated in this article, or claim that may be made by its manufacturer, is not guaranteed or endorsed by the publisher.
